# PMMA-Grafted Calcium Sulfate Whiskers for Applications as Fillers in PVC

**DOI:** 10.3390/polym14194199

**Published:** 2022-10-06

**Authors:** Qingbiao Li, Hao Liu, Chenchen Nie, Guiming Xie, Zhaomei Che, Dehui Zhu, Lei Guo, Yuan Xiang, Wei Shi

**Affiliations:** 1 School of Materials and Chemical Engineering, Tongren University, Tongren 554300, China; 2 School of Chemistry and Chemical Engineering, Guizhou University, Guiyang 550025, China; 3 Key Laboratory of Guizhou Province for Green Chemical Industry and Clean Energy Technology, Guizhou University, Guiyang 550025, China

**Keywords:** calcium sulfate whiskers, in situ polymerization, mechanical properties, polymer composites, polyvinyl chloride, surface modification

## Abstract

Calcium sulfate whiskers (CSWs) were hydroxylated with a sodium hydroxide (NaOH) solution and isolated for subsequent treatment with an ethanolic 3-(methacryloxy)propyltrimethoxysilane (KH570) solution to introduce C=C double bonds on the CSWs’ surfaces. Then, CSW-g-PMMA was prepared by grafting polymethyl methacrylate (PMMA) onto the surface of modified CSW using in situ dispersion polymerization. The CSW-g-PMMA was used as a filler and melt-blended with polyvinyl chloride (PVC) to prepare PVC-based composites. The surface chemical structure, PMMA grafting rate, and hydrophobic properties of CSW-g-PMMA were analyzed using X-ray diffraction, diffuse reflectance Fourier-transform infrared spectroscopy, thermogravimetric analysis, and water contact angle measurements, respectively. The effects of the CSW-g-PMMA filler on the mechanical properties of the CSW-PMMA/PVC composites were also investigated. The results showed that NaOH treatment significantly increased the number of hydroxyl groups on the surface of the CSWs, which facilitated the introduction of KH570. PMMA was successfully grafted onto the KH570 with a grafting rate of 14.48% onto the surface of the CSWs. The CSW-g-PMMA had good interfacial compatibility and adhesion properties with the PVC matrix. The tensile, flexural, and impact strengths of the CSW-g-PMMA/PVC composite reached 39.28 MPa, 45.69 MPa, and 7.05 kJ/m^2^, respectively, which were 38.55%, 30.99%, and 20.10% higher than those of the CSW/PVC composite and 54.52%, 40.80%, and 32.52% higher than those of pure PVC, respectively. This work provides a new method for surface modification of inorganic fillers, resource utilization, and high value-added application of CSWs from phosphogypsum.

## 1. Introduction

Calcium sulfate whiskers (CSWs) are single crystals with a large aspect ratio that have excellent properties such as good shape integrity, high strength, high temperature resistance, chemical resistance, and high toughness [1,2,3]. When used as reinforcing fillers, CSWs significantly improve the impact resistance, modulus, and heat resistance of polyvinyl chloride (PVC) composites [4,5]. In addition, CSWs are superior to many other whisker materials because they are non-toxic, resistant to chemical corrosion, structurally stable in size, easy modify via surface treatment, and have a high performance-to-price ratio [6,7]. However, this material has a high surface energy and is poorly compatible with the PVC matrix in composites. As a result, CSW fillers do not disperse well and easily agglomerate in the PVC matrix. This poor dispersion hinders the efficient transfer of externally applied stresses from the PVC matrix to the CSW filler and reduces the overall performance of composites [8,9,10].

The dispersion of the fillers can be improved via surface modification of the inorganic particles by increasing the surface tension and roughness of the particles or changing their surface chemical properties to improve the adhesion between the particles and the matrix [11,12,13,14,15,16,17,18,19,20]. For example, Yang et al. [21] modified nanostructured calcium carbonate (nano-CaCO_3_) with γ-(2,3-epoxypropyloxy)propyltrimethoxysilane (KH560), 3-(methacryloxy)propyltrimethoxysilane (KH570), or isopropyl dioleoyloxy(dioctyl phosphate acyloxy) titanate. The resulting rubber and modified nano-CaCO_3_ particles were refluxed in toluene to prepare core (nano-CaCO_3_)/shell rubber fillers that enhanced the rigidity of PVC composites.

Surface modification of inorganic filler particles is usually achieved via the reaction of hydroxyl groups on the particle surface with conventional filler particles such as titanates, aluminates, stearic acid (calcium), and silane coupling agents. The surfaces of several common inorganic fillers such as calcium carbonate and silicon dioxide are rich in a large number of hydroxyl groups, which can be effectively grafted with coupling agents for surface modification. However, the surface of CSWs contain fewer hydroxyl groups and the grafting rate to the surface of CSWs is very low. Surface hydroxylation is an effective method to increase the number of hydroxyl groups on the surface of CSWs and thereby significantly increase the grafting rate. Chen et al. [22] increased the number of hydroxyl groups on the surface of CaSO_4_ using a surface hydroxylation treatment, which then significantly increased the grafting rate of the surface modifier aminopropyltrimethoxysilane (APS). The surface modification significantly enhanced the interfacial interactions between the hydroxylated and APS-modified CaSO_4_ and the PVC matrix. As a result, the impact strength and flexural strength of the composite were increased by 13.7 kJ/m^2^ and 13.4 MPa, respectively, when compared with the CaSO_4_/PVC composite prepared with APS-modified CaSO_4_ without first performing the hydroxylation reaction. Lu et al. [23] successively used a sodium hydroxide (NaOH) solution and polyether titanate coupling agent to hydroxylate CSWs and then modify their surfaces, which significantly improved the interfacial compatibility between the CSWs and PVC.

Although conventional modification methods for inorganic filler particles overcome issues of low particle dispersion and insufficient interfacial compatibility between the filler and polymer matrix, the conventional small-molecule modifiers easily migrate from the interface to the surface of polymer composites. The van der Waals forces between typical modified inorganic filler particles and the polymer matrix are weak and the mechanical properties of the polymer composites are not improved significantly [24,25,26]. Studies have shown that increasing the alkyl chain length of the surface improves the interactions between the particles and the polymer matrix [27]. Therefore, several works proposed modifying inorganic filler particles with long-chain polymers [28,29,30]. Grafting such polymers on the particle surfaces has several advantages. First, it can improve the lipophilicity of inorganic particles, thereby improving the compatibility with the target polymer matrix. Second, the steric hinderance between the grafted polymer chains prevents the aggregation of the particles in the matrix. Third, the grafted polymer forms a transition layer that improves the binding between the grafted chains and the polymer matrix [31,32,33]. Shi et al. [34] filled PVC with poly(methyl methacrylate) (PMMA)-grafted CaCO_3_. The study found that the PMMA coating changed the surface properties of the particles and improved the bond strength between the particles and the PVC matrix. It was more difficult to disperse the fillers under mechanical shear and a tensile force. Furthermore, the modified particles were better ‘embedded’ in a PVC matrix. Cui et al. [35] grafted PMMA onto the surface of CaCO_3_ whiskers using an in situ emulsion polymerization and used the obtained material as a filler in PVC. The tensile strength of the PVC composite increased and the modified CaCO_3_ whiskers demonstrated a strong interfacial adhesion to the PVC matrix. Phosphogypsum is an industrial solid waste produced in large quantities during the wet processing of phosphoric acid and pollutes the environment [36]. Preparing CSWs from phosphogypsum is a promising strategy to create a value-added product from industrial solid waste [37]. Although there are many studies on the modification of CSWs with small molecules, we found no reports on the modification of CSWs with long-chain polymers such as PMMA, or the use of polymer-modified CSWs as fillers in polymer composites.

In this study, an aqueous NaOH solution was used to treat CSWs to form a calcium hydroxide (Ca(OH)_2_) layer on the CSWs’ surfaces to increase the number of surface hydroxyl groups. Then, C=C double bonds were introduced via modification with KH570 and the PMMA was grafted onto the modified CSW surface using in situ polymerization. The dispersion of modified CSWs in a PVC matrix was improved and the interface between the CSWs and the PVC matrix was enhanced to improve the mechanical properties of the PVC/CSW composites. The surface chemical structure, PMMA grafting rate, and surface hydrophobicity of the modified CSWs were analyzed using X-ray diffraction (XRD), diffuse reflectance Fourier-transform infrared spectroscopy (DRIFTS), field-emission scanning electron microscopy (FE-SEM), thermogravimetric analysis (TGA), and water contact angle measurements. The effect of CSW surface modification on the mechanical and thermal properties of the PVC/CSW composites was studied.

## 2. Materials and Methods

### 2.1. Materials

The CSWs were purchased from Jiangsu Xinyuan Mining Co., Ltd., Yixing, China. The KH570 (analytically pure) was purchased from Changzhou Runxiang Chemical Co., Ltd., Changzhou, China. The NaOH (analytically pure) was bought from Chongqing Chuandong Chemical Group Co., Ltd., Chongqing, China. The anhydrous sodium sulfate (Na_2_SO_4_, analytically pure) and dioctyl phthalate (DOP, analytically pure) were supplied by Tianjin Zhiyuan Chemical Reagent Co., Ltd., Tianjin, China. The anhydrous ethanol (analytically pure) and isopropanol (analytically pure) were purchased from Tianjin Fuyu Fine Chemical Co., Ltd., Tianjin, China. The polyvinylpyrrolidone (PVP, excellent-grade pure) was bought from Chemical Reagent Co., Ltd., Shanghai, China; and the benzoyl peroxide (BPO, analytically pure) from Shanghai McLin Biochemical Technology Co., Ltd., Shanghai, China. The methyl methacrylate (MMA, chemically pure) was purchased from Shanghai Lingfeng Chemical Reagent Co., Ltd., Shanghai, China. The PVC resin (SG-05, injection grade) was supplied by Inner Mongolia Wuhai Chemical Co., Ltd., Inner Mongolia, China. The calcium/zinc (Ca/Zn) stabilizer (8021, industrial grade) was purchased from Dongguan Shangnuo New Materials Co., Ltd., Guangdong, China. The lubricant polyethylene (PE) wax (industrial grade) was purchased from Sichuan Jinsen Plastic Co., Ltd., Sichuan, China. The brightener (TAS-2A, industrial grade) was bought from Suzhou Xingtai Photochemical Additives Co., Ltd., Xingtai, China. The ACR8901 plasticizer (industrial grade) was purchased from Shijiazhuang Huake Chemical Co., Ltd., Hebei, China; and the CPE135A plasticizer (industrial grade) from Chengdu Wante Plastics Co., Ltd., Sichuan, China. The deionized water was prepared using a Milli-Q water purification system from Merck, Sichuan, China.

### 2.2. Experimental Methods

#### 2.2.1. CSW Hydroxylation and KH570 Modification

At room temperature, 70 g of CSWs, 30 g of NaOH, and 5 g of Na_2_SO_4_ (to inhibit hydration and dissolution of the CSWs) were added to 300 g of deionized water. After stirring for 60 min, the mixture was filtered and the filter cake was washed with deionized water and dried under a vacuum at 55 °C for 12 h to obtain the hydroxylated CSWs (CSW-OH). A volume of 150 mL of anhydrous ethanol, 7.5 mL of deionized water, and 3.2 g of KH570 were placed into a three-necked flask equipped with a reflux condenser. The flask was purged with nitrogen to remove the air. After heating to 60 °C under stirring, 30 g of CSW-OH was added. After a certain reaction time, the mixture was filtered, washed three times with anhydrous ethanol, and dried under a vacuum at 55 °C for 12 h to obtain the modified CSW material (CSW-OH-KH570).

#### 2.2.2. Preparation of CSW-g-PMMA Using In Situ Dispersion Polymerization

A mass of 7 g of CSW-OH-KH570 was added to 200 mL of isopropyl alcohol containing 1.0 g of dissolved PVP. After ultrasonic treatment for 10 min, the mixture was transferred into a four-necked flask equipped with a reflux condenser, a thermometer, an agitator, and a nitrogen inlet. The subsequent reactions were performed under inert gas conditions. After heating to 70 °C, a solution of 0.2 g of BPO in 10 mL of isopropyl alcohol was added. After 15 min of stirring at this temperature, a solution of 10 g of MMA in 20 mL of isopropyl alcohol was slowly dropped into the flask via a pressure-equalizing dropping funnel for 1 h. After a reaction time of 10 h, the mixture was filtered, washed with deionized water and isopropyl alcohol, and dried in an oven. Using acetone as solvent, the product was extracted in a Soxhlet extractor for 48 h to remove the PMMA homopolymer from the product and obtain the PMMA-grafted CSWs (CSW-g-PMMA).

#### 2.2.3. Preparation of PVC Composites via Melt Blending of PVC Filled with CSW Materials

**Figure 1:**
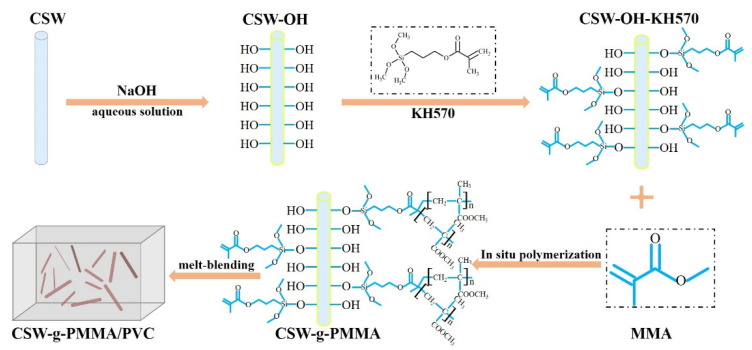
Schematic diagram of PMMA-grafted CSWs for application as fillers in PVC.

The samples were prepared by evenly mixing 12 g of filler (either untreated CSWs, CSW-OH, CSW-OH-KH570, or CSW-g-PMMA) with 300 g of PVC resin, 18 g of Ca/Zn stabilizer, 70 g of DOP, 1.5 g of PE wax, 1.5 g of brightener, 21 g of ACR8901, and 6 g of CPE135A. Each combination was mixed in a rubber plastic test mixer (XSM-500, Shanghai Science and Technology Rubber and Plastic Machinery Equipment Co., Ltd., Shanghai, China) at a rotor speed of 80 r/min and a temperature of 185 °C for 10 min. After a sample was cooled, it was crushed into particles using a strong crusher (PC-250, Shantou Taisheng Plastic Machinery Co., Ltd., Shantou, China). Then, the obtained sample was used in an injection molding machine (PL860, Wuxi Haitian Machinery Co., Ltd., Wuxi, China) operated according to the spline shapes specified in the ISO 527, ISO 178, and ISO 179 standards. Figure 1 shows a schematic diagram of the PMMA-grafted CSWs prepared here as fillers for PVC.

### 2.3. Characterization Methods

X-ray diffraction (XRD, D8 Advance, Bruker, Billerica, MA, USA) was used to determine sample phases using Cu Kα radiation (λ = 1.5418 Å), a scanning speed of 10°/min, and a scanning range between 10° and 80°. The functional groups present in the samples were determined using diffuse reflectance infrared Fourier-transform spectroscopy (DRIFTS, Nicolet iS50, Thermo Fisher Scientific, Waltham, MA, USA). DRIFTS data were collected in the spectral range of 500–4000 cm^−1^; the presented spectra are the average of 64 scans. The morphology of the samples was observed via scanning electron microscopy (SEM, ΣSIGAIGMA + X-Max 20, Zeiss, Oberkochen, Germany). The test voltage range was 0.02–30 kV and the pressure was between 2 and 133 Pa. The samples were sprayed with gold before the test. A thermogravimetric analyzer (TGA, 209F1Libra, Netzsch, Weimar, Germany) was used to determine the thermogravimetric (TG) curve of the sample. The tests were conducted in a nitrogen atmosphere; the temperature was raised from room temperature to 650 °C at a heating rate of 10 °C/min. The grafting rate of the KH570 on the CSWs was calculated using Equation (1):

(1) $$\mathrm{Grafting}\;\mathrm{rate}=\frac{W_{A}-W_{B}}{W_{C}}\times 100\%$$

where *W*_A_ is the weight loss of the CSW-OH-KH570 from 50 °C to 600 °C, *W*_B_ is the weight loss of the CSW-OH from 50 °C to 600 °C, and *W*_C_ is the residual weight of the CSW-OH-KH570 at 600 °C. The grafting rate of the PMMA in the CSW-g-PMMA on the surface of the KH570-modified CSWs was determined using TGA and calculated according to Equation (2):

(2) $$\mathrm{Grafting}\;\mathrm{rate}=\frac{W_{D}-W_{A}}{W_{E}}\times 100\%$$

where *W*_D_ is the weight loss of the CSW-g-PMMA from 50 °C to 600 °C, *W*_A_ is the weight loss of the CSW-OH-KH570 from 50 °C to 600 °C, and *W*_E_ is the residual weight of the CSW-g-PMMA at 600 °C.

A total of 3 g of the sample was pressed into a wafer using a tablet press (HDRY-40, Shanghai Hengzhan Industrial Co., Ltd., Shanghai, China) and the water contact angle of the sample was measured using a contact angle tester (SDC-100, China Shengding Precision Instrument Co., Ltd., Shanghai, China). First, five evenly distributed points were selected on the sample surface, then deionized water droplets measuring 3 μL were dropped on these five points using the sampling needle of the instrument. The corresponding contact angle values were read. Finally, the average value of the five contact angles was calculated.

The mechanical properties of the composites were tested using a microcomputer-controlled electronic universal testing machine (TSE104B, Shenzhen Universal Testing Equipment Co., Ltd., Shenzhen, China) and the tensile properties were tested according to the ISO 527 standard. The three-point bending mode was adopted to conduct bending performance tests according to the ISO 178 standard. Each group of samples was subjected to five independent parallel measurements and the average result was taken. The impact performance was measured according to ISO 179, for which the sample was prepared using a notched sample machine (XQZ-II, Chengde Wan Plastic Testing Instrument Co., Ltd., Chengde, China). A liquid crystal pendulum impact tester (XJJ-5.5, Chengde Wan Plastic Testing Instrument Co., Ltd., Chengde, China) was used for testing. Five independent parallel measurements were conducted for each group of samples and the average value was calculated and reported.

## 3. Results and Discussion

### 3.1. Properties of Modified CSWs

**Figure 2:**
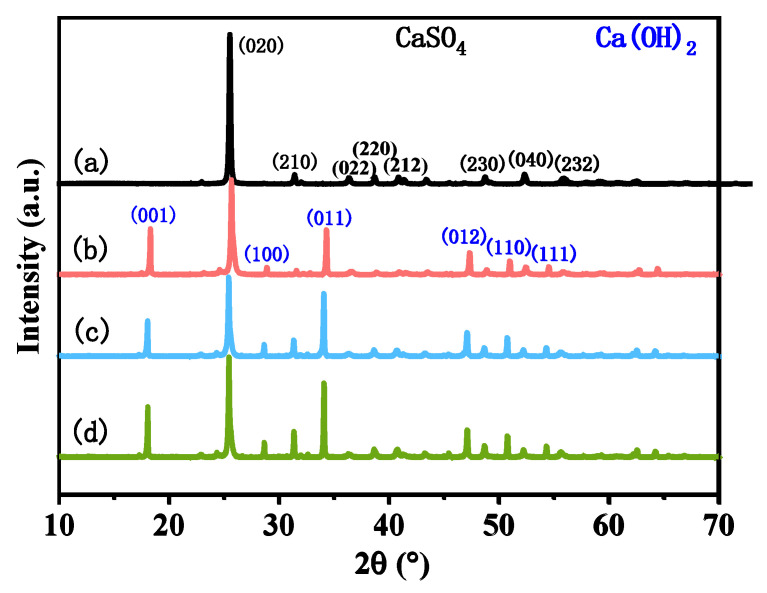
XRD patterns of (**a**) untreated CSWs, (**b**) CSW-OH, (**c**) CSW-OH-KH570, and (**d**) CSW-g-PMMA.

Figure 2 displays the XRD patterns of the untreated CSWs, CSW-OH, CSW-OH-KH570, and CSW-g-PMMA. As shown in Figure 2a, the untreated CSWs were type II-CaSO_4_ (PDF#74-2421, space group: Bbmm) and the corresponding 2θ values of 23.1°, 31.5°, 36.6°, 38.9°, 41.1°, 49.0°, 52.5°, and 56.1° were the main diffraction peaks of the (020), (210), (022), (220), (212), (230), (040), and (232) crystal planes of the type II-CaSO_4_, respectively. The reflection peak of the (020) crystal plane was the strongest. Compared with the peaks shown in Figure 2a, new peaks can be observed in Figure 2b at the 2θ values of 18.3°, 28.9°, 34.3°, 47.3°, 51.0°, and 54.4°, which corresponded to the (001), (100), (011), (012), (110), and (111) diffraction peaks of Ca(OH)_2_, respectively. Note that the diffraction peak intensity of the (020) CSW crystal plane was significantly reduced, which was attributed to the reaction between NaOH and CaSO_4_. The hydroxylation reaction resulted in the deposition of Ca(OH)_2_ on the surface of the CSWs, which reduced the amount of CaSO_4_ on the particle surface while significantly increasing the number of hydroxyl groups present on the surface of the obtained CSW-OH sample. The XRD patterns of the CSW-OH-KH570 and CSW-g-PMMA (Figure 2c,d) were not significantly different from those of the CSW-OH (Figure 2b), indicating that the type II-CaSO_4_ in the CSWs did not react with water during the KH570 modification reaction or the in situ graft polymerization of the PMMA and that the crystal form and internal structure of the CSWs did not change.

**Figure 3:**
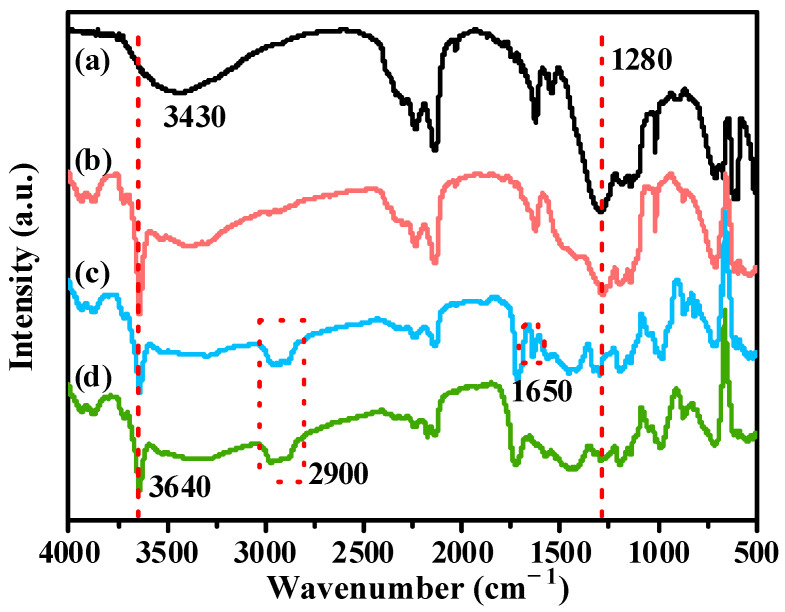
DRIFT spectra of (**a**) untreated CSWs, (**b**) CSW-OH, (**c**) CSW-OH-KH570, and (**d**) CSW-g-PMMA.

Figure 3 shows the DRIFT spectra of the untreated CSWs, CSW-OH, CSW-OH-KH570, and CSW-g-PMMA. In the DRIFT spectrum for the CSWs (Figure 3a), a broad peak was observed at 3430 cm^−1^, which indicated that hydrogen bonds formed between the hydroxyl groups and other molecules on the surfaces of CSWs. Due to the decrease in the system energy, the stretching vibration peaks for these hydroxyl groups shifted to a lower wavenumber of 3430 cm^−1^ from 3640 cm^−1^. Therefore, the intensity of the stretching vibration peak at 3640 cm^−1^, which is typical for surface hydroxyl groups, was very low, which confirmed that the number of hydroxyl groups on the surface of the untreated CSWs was small. Compared with the untreated CSWs, the DRIFT spectrum for the CSW-OH (Figure 3b) contained a strong hydroxyl stretching vibration peak at 3640 cm^−1^, indicating that the number of hydroxyl groups on the surface of the CSWs was significantly increased after hydroxylation treatment. Simultaneously, the antisymmetric stretching vibration peak of SO_4_^2−^ at 1280 cm^−1^ appeared to be relatively weakened, which was attributed to the reaction of NaOH with the CSWs to generate Ca(OH)_2_ and the loss in SO_4_^2−^, which corresponded to the new Ca(OH)_2_ diffraction peak in the XRD spectrum shown in Figure 2b and the weakening of the main diffraction peak of the type II-CaSO_4_ crystal plane (020). Compared with those of the untreated CSWs and the CSW-OH, the DRIFT spectra of the CSW-OH-KH570 featured a C=C vibration peak at 1650 cm^−1^ and the antisymmetric and symmetric stretching vibration peaks for -(CH_2_)_n_- at 2900 cm^−1^, indicating that the KH570 was successfully grafted onto the surface of the CSWs. While the asymmetric and symmetric stretching vibration peaks for -(CH_2_)_n_- at 2900 cm^−1^ were also observed for the CSW-g-PMMA, the C=C vibration peak at 1650 cm^−1^ was absent [38], indicating that MMA and the C=C double bonds of KH570 on the surface of the CSWs reacted successfully in the grafting process during the in situ polymerization.

**Figure 4:**
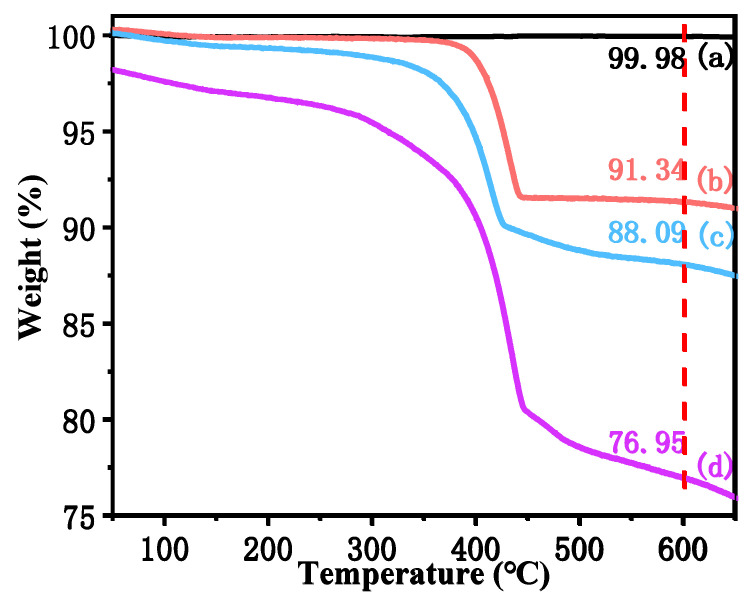
The thermogravimetric curves for (**a**) untreated CSWs, (**b**) CSW-OH, (**c**) CSW-OH-KH570, and (**d**) CSW-g-PMMA.

Figure 4 shows the thermogravimetric curves of for the untreated CSWs, CSW-OH, CSW-OH-KH570, and CSW-g-PMMA. In Figure 4a, the weight loss of the untreated CSWs was less than 0.02% at 600 °C. According to Figure 4b, the CSW-OH had a high weight loss rate of between 350 and 600 °C and a weight loss of 9.66% at 600 °C. The weight loss in this temperature range was attributed to the decomposition of Ca(OH)_2_ on the CSW-OH surface that was not involved in the reaction [39], corroborating the existence of Ca(OH)_2_ on the CSW-OH surface after hydroxylation. As shown in Figure 4c, the weight loss of the CSW-OH-KH570 at 600 °C was 11.91% due to the weight loss of unreacted Ca(OH)_2_ on the CSW-OH-KH570 surface and organic chains of grafted KH570. The grafting rate of KH570 on the CSWs’ surface was calculated as 3.69% using Equation (1). In Figure 4d, the weight loss of the CSW-g-PMMA was 23.05% at 600 °C. The corresponding grafting ratio of the PMMA as calculated using Equation (2) was 14.48%, which indicated that the PMMA polymer was successfully grafted onto the CSWs’ surfaces with a high grafting ratio.

**Figure 5:**
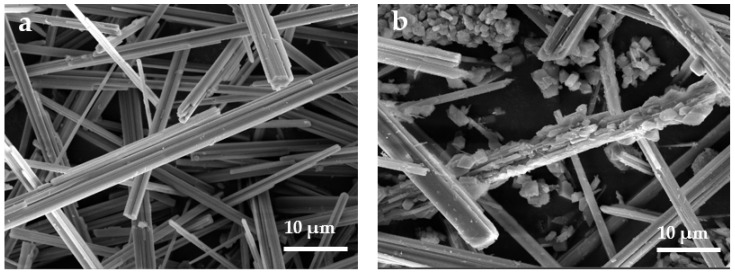

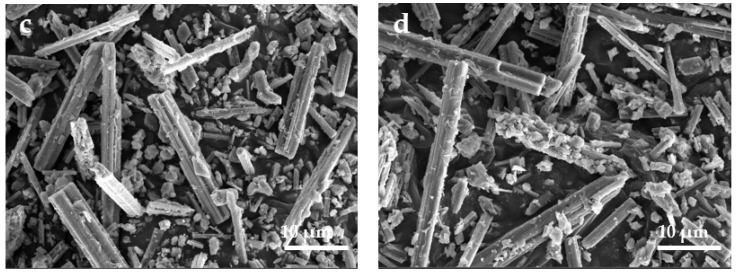
SEM images of (**a**) CSW, (**b**) CSW-OH, (**c**) CSW-OH-KH570, and (**d**) CSW-g-PMMA.

SEM images of the untreated CSWs, CSW-OH, CSW-OH-KH570, and CSW-g-PMMA are displayed in Figure 5. As can be seen in Figure 5a, the untreated CSWs were needle-like and their surfaces were very smooth. In contrast, the surface of the CSW-OH sample shown in Figure 5b is very rough, indicating the reaction of Na_2_SO_4_ with NaOH on the surface of the CSWs following the chemical equilibrium in Equation (3). Therefore, a Ca(OH)_2_ layer was deposited on the surface of the CSWs and longitudinal grooves were formed, leading to a rougher surface. Figure 5c shows that the surface morphology of the CSW-OH-KH570 sample was similar to that of the CSW-OH and that the surface of the CSW-OH-KH570 sample became rougher due to the grafting of the KH570 onto the surface of the CSW-OH sample. As shown in Figure 5d, the roughness of the CSW-g-PMMA’s surface further increased due to the long-chain PMMA polymer coating.

(3) $${\mathrm{CaSO}}_{4}+2\;\mathrm{NaOH}\;⇌\;{\mathrm{Na}}_{2}{\mathrm{SO}}_{4}+\mathrm{Ca}{\left(OH\right)}_{2}$$

**Figure 6:**
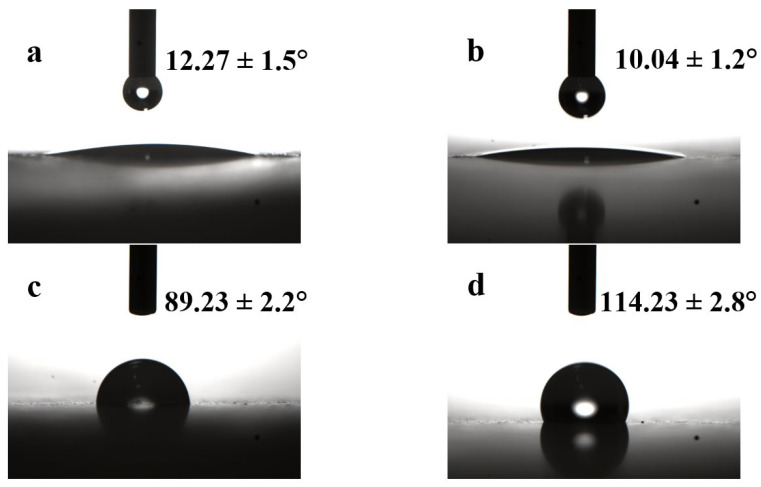
The water contact angles on the surfaces of the (**a**) untreated CSWs, (**b**) CSW-OH, (**c**) CSW-OH-KH570, and (**d**) CSW-g-PMMA.

Figure 6 shows the water contact angles on the surfaces of the untreated CSWs, CSW-OH, CSW-OH-KH570, and CSW-g-PMMA. The contact angles of the untreated CSWs and CSW-OH were 12.27° and 10.04°, respectively, indicating that the surfaces were hydrophilic and hydroxylation did not change the polarity of the CSWs’ surfaces. Due to the increase in the number of surface hydroxyl groups, the CSW-OH surface was more hydrophilic. As shown in Figure 6c, the water contact angle of the CSW-OH-KH570 was 89.23° because the long organic KH570 chains were outward-oriented and made the particle surface more hydrophobic. Figure 6d shows that the water contact angle of the CSW-g-PMMA sample was 114.23°, which was significantly higher than that of the untreated CSWs and the CSW-OH sample and 25° higher than that of the CSW-OH-KH570 sample. The surface hydrophobicity of the CSW-g-PMMA sample was significantly increased because, after in situ polymerization of the CSW-OH-KH570 and MMA, long PMMA polymer chains were linked to the surfaces of the CSWs and effectively covered the entire surface.

### 3.2. Properties of PVC Composites

#### 3.2.1. Mechanical Properties

**Figure 7:**
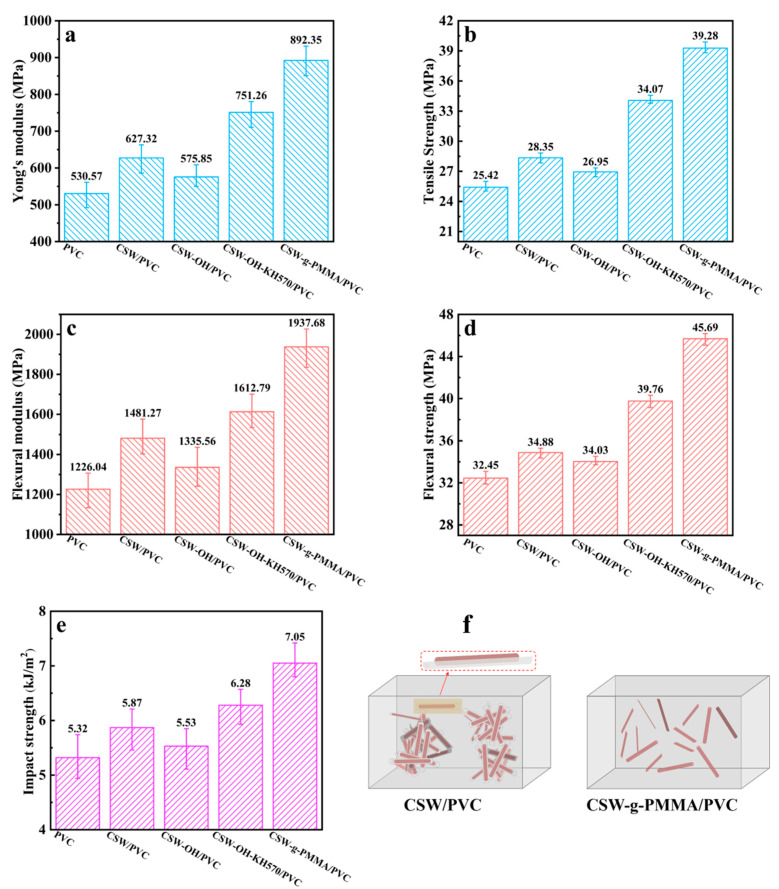
The mechanical properties of (**a**) Young’s modulus, (**b**) tensile strength, (**c**) flexural modulus, (**d**) flexural strength, and (**e**) impact strength for pure PVC and composites of untreated CSW/PVC, CSW-OH/PVC, CSW-OH-KH570/PVC, and CSW-g-PMMA/PVC, respectively; (**f**) schematic diagram of the dispersion of untreated CSWs and CSW-g-PMMA in the PVC matrix, respectively.

Figure 7 provides information on the Young’s modulus, tensile strength, flexural modulus, flexural strength, and impact strength of the pure PVC and the untreated CSW/PVC, CSW-OH/PVC, CSW-OH-KH570/PVC, and CSW-g-PMMA/PVC composites. In Figure 7a, it can be seen that the Young’s modulus values for all of the PVC composites were much higher than those of the pure PVC material, which indicated that the addition of the CSWs stiffened the composite regardless of whether the surfaces of the whiskers were modified. However, the Young’s modulus values for the CSW-OH-KH570/PVC and CSW-g-PMMA/PVC composites were higher than those of the untreated CSW/PVC and CSW-OH/PVC composites. These results showed that the surface modification of the CSWs by KH570 and PMMA improved the interfacial compatibility between the filler and matrix so that the CSW fillers reinforced the PVC matrix. In Figure 7b, the tensile strength of pure PVC, untreated CSW/PVC, CSW-OH/PVC, CSW-OH-KH570/PVC, and CSW-g-PMMA/PVC were 25.42 MPa, 28.35 MPa, 26.95 MPa, 34.07 MPa, and 39.28 MPa, respectively. The tensile strengths of the PVC composites with CSW fillers were higher than that of pure PVC regardless of whether the CSWs were modified. Because CSWs are a needle-like single-crystal material with a large aspect ratio, these fillers could bear a part of the external force exerted on the PVC composite. The tensile strength of the CSW-g-PMMA/PVC was the highest, followed by the CSW-OH-KH570/PVC, because the hydrophobic properties of the CSWs’ surfaces increased with an increasing chain length of the grafted organic modifier and the PMMA chains were longer than the KH570. These long organic chains could penetrate into the PVC matrix and enhance the adhesion and interface between the CSWs and the PVC matrix. The tensile strength of the CSW-OH/PVC composite was lower than that of the untreated CSW/PVC composite due to the higher number of hydroxyl groups on the surface of the CSW-OH, which resulted in an increase in polarity and surface hydrophilicity (as shown in Figure 6a,b). Therefore, the compatibility between the CSW-OH and the PVC matrix was poor, leading to poor stress transfer at the interface between the CSW filler and PVC matrix [40,41].

As can be seen in Figure 7c, the flexural modulus values for all of the PVC composites were higher than that for pure PVC, indicating that the CSW filler increased the stiffness of the composites. This enhancement occurred because the elastic moduli of the CSW fillers were higher than that of the PVC material. Under small applied strains that were within the elastic deformation range, the rigid CSW filler bore part of the external force such that the modulus of the composite was improved overall. As shown in Figure 7d, the flexural strength of the pure PVC, untreated CSW/PVC, CSW-OH/PVC, CSW-OH-KH570/PVC, and CSW-g-PMMA/PVC were 32.45 MPa, 34.88 MPa, 34.03 MPa, 39.76 MPa, and 45.69 MPa, respectively. The flexural strength of the CSW-OH-KH570/PVC composite was the highest, followed by the CSW-g-PMMA/PVC, and was much higher than those of the pure PVC, untreated CSW/PVC, and CSW-OH/PVC. Compared with the pure PVC and the CSW/PVC composite, the flexural strength of the CSW-g-PMMA/PVC increased by 40.80% and 30.99% and the flexural strength of the CSW-OH-KH570/PVC increased by 22.53% and 13.99%, respectively. This increase in strength was attributed to the hydrophobicity of the CSW-g-PMMA and CSW-OH-KH570 fillers and the more even dispersion of the modified fillers in the PVC matrix. The organic segments of the PMMA polymer and KH570 significantly enhanced the adhesion and interfacial interaction between the CSWs and the PVC matrix [21]. Figure 7e shows that the impact strength of the pure PVC and the untreated CSW/PVC, CSW-OH/PVC, CSW-OH-KH570/PVC, and CSW-g-PMMA/PVC composites were 5.32 kJ/m^2^, 5.87 kJ/m^2^, 5.53 kJ/m^2^, 6.28 kJ/m^2^, and 7.08 kJ/m^2^, respectively. Whether the CSW was modified or not, the addition of a filler improved the impact resistance of the composite by contributing to the absorption and transfer of the impact energy. Thus, the impact resistance of the PVC composites was improved. In addition, with an increasing hydrophobicity of the CSWs’ surfaces and an increasing length of the grafted organic chain segment, the adhesion and interface between the CSWs and the PVC matrix became stronger and the impact strength of the PVC composites increased. Therefore, the impact strength of the CSW-g-PMMA/PVC composite was the largest (20.1% higher than that of the untreated CSW/PVC composite). As shown in the schematic diagrams of the untreated CSW/PVC and CSW-g-PMMA/PVC composites in Figure 7f, the CSW filler agglomerated in the untreated CSW/PVC composite; a gap between the CSW filler and the PVC matrix can be observed in the enlarged image. After the KH570 modification and the PMMA-grafting process, the CSW filler in the CSW-g-PMMA/PVC composite was uniformly dispersed in the PVC matrix and no interfacial gaps were seen. These results showed that KH570 modification followed by PMMA grafting resulted in an overall improvement in the properties of the CSW-g-PMMA/PVC composite.

#### 3.2.2. Section Morphology

**Figure 8:**
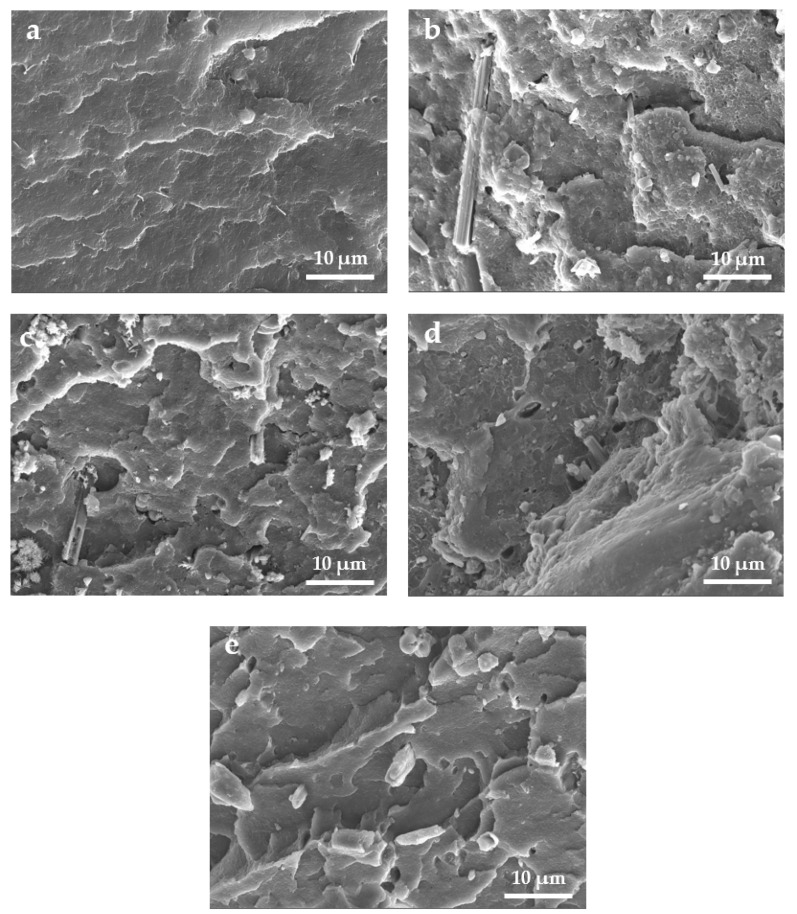
Section morphology of (**a**) pure PVC, (**b**) untreated CSW/PVC, (**c**) CSW-OH/PVC, (**d**) CSW-OH-KH570/PVC, and (**e**) CSW-g-PMMA/PVC.

Figure 8 displays the SEM images of the pure PVC, untreated CSW/PVC, CSW-OH/PVC, CSW-OH-KH570/PVC, and CSW-g-PMMA/PVC quenched in liquid nitrogen. As shown in Figure 8a, the cross-section of the pure PVC material was relatively smooth, indicating that the crack propagation of the pure PVC was a relatively easy fracturing process. As shown in Figure 8b,c, voids were generated by CSW pulling out of the PVC matrix during fracture. This observation can be explained by the relatively smooth and hydrophilic surfaces of the CSWs and CSW-OH, which were poorly compatible with the PVC matrix. As a result, the interfacial interactions were weak and the applied stress on the material could not be fully transferred from the PVC matrix to the filler. However, after hydroxylation and KH570 modification, the hydrophobicity of the CSW-OH-KH570 was higher. The organic KH570 segments present on the filler surface significantly improved the interfacial compatibility between the CSW-OH-KH570 and the PVC matrix, resulting in closer adhesion of the two materials, as shown in Figure 8d. According to Figure 8e, there were no obvious gaps between the CSWs and the matrix in the CSW-g-PMMA/PVC composite. CSWs were tightly embedded in the PVC matrix, indicating that the interactions between the CSWs and the matrix were strong and that the filler and matrix were in close contact and strongly adhered. Although some CSWs disintegrated in the CSW-g-PMMA/PVC composite during liquid-nitrogen quenching, the applied stress could still be effectively transferred between the two phases.

#### 3.2.3. Thermal Stability

**Figure 9:**
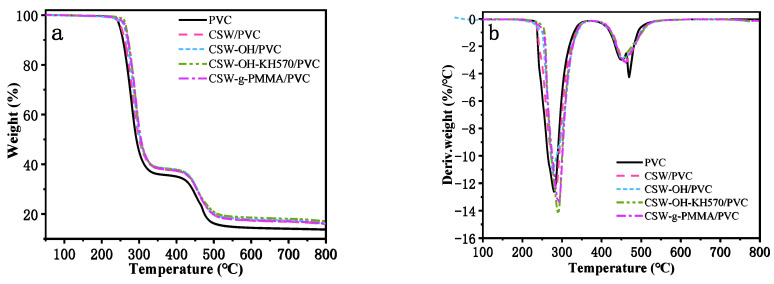
The thermogravimetric curves (**a**) and derivative thermogravimetric curves (**b**) of pure PVC, untreated CSW/PVC, CSW-OH/PVC, CSW-OH-KH570/PVC, and CSW-g-PMMA/PVC.

The thermogravimetric and derivative thermogravimetric curves for the pure PVC and the untreated CSW/PVC, CSW-OH/PVC, CSW-OH-KH570/PVC, and CSW-g-PMMA/PVC composites are provided in Figure 9. As reported previously, two weight-loss stages were observed for the pure PVC and its composites. The first weight-loss stage occurred between 230 °C and 420 °C, which corresponded to the removal of hydrogen chloride (HCl) from the PVC materials via dehydrochlorination under the formation of C=C double bonds in the polymer chain [42]. After removing the HCl, the pure PVC formed conjugated double bonds and again achieved thermal stability [43]. The second weight-loss stage occurred in the temperature range of 420–535 °C, corresponding to the pyrolysis of polyacetylene (breaking of covalent bonds and multi-bonds) [44], and then stable carbon black residue was formed at temperatures higher than 473 °C. The initial decomposition temperature (*T*_onset_), maximum weight loss rate temperature (*T*_rpd_), and 50% weight loss residue temperature (*T*_50_) of the PVC composites are provided in Table 1. The *T*_onset_, *T*_rpd_, and *T*_50_ of the CSW-g-PMMA/PVC were the highest, followed by CSW-OH-KH570/PVC, and all temperatures were higher than those for pure PVC, untreated CSW/PVC, and CSW-OH/PVC, indicating that the addition of CSWs could improve the thermal stability of the PVC composites regardless of whether the CSWs were pristine or modified. The increase in surface hydrophobicity of the CSWs, which was caused by longer organic chains covering the surface, was more conducive to enhancing the adhesion and interfacial interaction between the CSWs and the PVC matrix [45] and improving the thermal stability of the PVC composites. Therefore, the CSW-g-PMMA/PVC composite exhibited increases in the *T*_onset_ and *T*_rpd_ of 16 and 14 °C, respectively, compared with the pure PVC.

This work greatly improved the grafting rate of the polymer on the CSWs. However, the experimental process was relatively complicated and the CSW modification procedure required three steps. In the future, grafting the initiator directly to the CSWs to initiate the polymerization would greatly reduce the number of steps required for the polymerization reaction.

## 4. Conclusions

In summary, a surface-modification method for CSW was proposed to overcome the incompatibility between bare CSWs and a PVC polymer matrix, and the following conclusions were drawn:

(1) The surfaces of CSWs were hydroxylated in an aqueous NaOH solution to increase the number of surface hydroxyl groups. Subsequent introduction of KH570 containing C=C double bonds enabled surface-initiated polymerization. Long-chain PMMA polymers were successfully grafted on the surface of CSWs via in situ polymerization, which made the CSWs more hydrophobic. The water contact angle increased from 12.27° for the bare CSWs to 89.23° for the CSW-OH-K570 and 114.23° for the CSW-g-PMMA.
(2) The CSW-g-PMMA/PVC composites prepared with the long-chain-PMMA grafted filler exhibited better mechanical properties. The tensile strength, flexural strength, and impact strength reached 39.28 MPa, 45.69 MPa, and 7.05 kJ/m^2^, respectively, which were 15.29%, 14.92%, and 12.26% higher than that of the CSW-OH-KH570/PVC composite, respectively.
(3) The CSW-g-PMMA/PVC composite showed the best thermal stability, followed by the CSW-OH-KH570/PVC composite. Both composites prepared with the surface-modified CSWs were more thermally stable than the pure PVC, untreated CSW/PVC, and CSW-OH/PVC composites.

This work provided a new method for the surface modification of inorganic fillers as well as improved resource utilization and a high value-added application of CSWs from phosphogypsum.

## Figures and Tables

**Table 1:** Decomposition stability of PVC composites.

Sample	Temperature (°C)		
	*T*_onset_	*T*_rpd_	*T*_50_
Pure PVC	229	279	292
Untreated CSW/PVC	233	283	300
CSW-OH/PVC	231	280	302
CSW-OH-KH570/PVC	243	290	304
CSW-g-PMMA/PVC	245	293	305

## Data Availability

Not applicable.

## Nomenclature

CSWs	Calcium sulfate whiskers
KH570	3-(Methacryloxy)propyltrimethoxysilane
PMMA	Polymethyl methacrylate
PVC	Polyvinyl chloride
nano-CaCO_3_	Nanostructured calcium carbonate
KH560	γ-(2,3-epoxypropyloxy)propyltrimethoxysilane
APS	Aminopropyltrimethoxysilane
MMA	Methyl methacrylate
DOP	Dioctyl phthalate
ACR8901	Impact modifier
CPE135A	Chlorinated polyethylene 135A
PVP	Polyvinylpyrrolidone
BPO	Benzoyl peroxide
PE	Polyethylene
CSW-OH	Hydroxylated calcium sulfate whisker
CSW-OH-KH570	KH570-modified calcium sulfate whisker
CSW-g-PMMA	Polymethyl methacrylate-grafted calcium sulfate whisker
*W*_A_	Weight loss of CSW-OH-KH570 from 50 °C to 600 °C (%)
*W*_B_	Weight loss of CSW-OH from 50 °C to 600 °C (%)
*W*_C_	Residual weight of CSW-OH-KH570 at 600 °C (%)
*W*_D_	Weight loss of CSW-g-PMMA from 50 °C to 600 °C (%)
*W*_E_	Residual weight of CSW-g-PMMA at 600 °C (%)
*T*_onset_	Decomposition temperature
*T*_rpd_	Maximum weight loss rate temperature
*T*_50_	50% weight loss residue temperature
